# Promise and unrealized potential: 10 years of the American Medical Association classifying obesity as a disease

**DOI:** 10.3389/fpubh.2023.1205880

**Published:** 2023-07-14

**Authors:** Leah M. Schumacher, Jamy Ard, David B. Sarwer

**Affiliations:** ^1^Department of Kinesiology, College of Public Health, Temple University, Philadelphia, PA, United States; ^2^Center for Obesity Research and Education, College of Public Health, Temple University, Philadelphia, PA, United States; ^3^Department of General Surgery, Atrium Health Wake Forest Baptist, Weight Management Center, Winston-Salem, NC, United States; ^4^Department of Epidemiology and Prevention, Wake Forest School of Medicine, Winston-Salem, NC, United States; ^5^Department of Social and Behavioral Sciences, College of Public Health, Temple University, Philadelphia, PA, United States

**Keywords:** obesity, policy, treatment, prevention, access, health equity (MeSH), weight stigma

## Abstract

**Introduction:**

In June 2013, the American Medical Association (AMA), one of the most influential healthcare organizations in the United States, voted to recognize obesity as a disease. Many who supported this change believed that recognition by AMA of obesity as a disease would serve as a tipping point that would increase access to care, accelerate training and research on the prevention and treatment of obesity, and reduce weight stigma. On the 10-year anniversary of this vote, this perspective piece outlines key advances made, as well as unrealized potential, in improving the obesity public health landscape since the AMA’s classification of obesity as a disease.

**Methods:**

We draw on the empirical literature, as well as our experiences as clinical psychologists, a physician, and public health researchers specializing in obesity, to provide an overview of major advances and continued challenges in improving access to obesity treatment, accelerating prevention and training, and reducing weight stigma. We also outline important next steps to advance these goals.

**Results:**

While several notable advancements have occurred, significant work remains to create equitable access to evidence-based treatments, bring research and training on obesity on par with its prevalence, and reduce the pervasiveness and harm of weight stigma.

**Conclusion:**

The past decade has witnessed some advances with respect to access to care and attention, yet there is unrealized potential that awaits attention. Truly conceptualizing and treating obesity as a chronic disease requires a major paradigm shift.

## 1. Introduction

Obesity is a significant public health issue in the United States. More than 42% of American adults and 20% of American youth have obesity (1). Another 30% of adults and 16% of youth are classified as overweight, placing them at increased risk of developing obesity in the future (2, 3). The impact of obesity is notable. Obesity is associated with increased risk for numerous diseases and premature morbidity, poorer health-related quality of life, and significant economic costs (1, 4, 5). The prevalence and costs of obesity are only expected to grow in the coming years. For example, it is predicted that over one half of the world’s population will have overweight or obesity by 2035 and the economic impact will exceed $4 trillion (1).

In June 2013, the American Medical Association (AMA) House of Delegates voted to recognize obesity as a disease. While the AMA was not the first organization to classify obesity as a disease, this decision was significant as the AMA is one of the most influential healthcare organizations in the United States. Many who advocated for and supported this change believed that recognition by AMA of obesity as a disease would serve as a tipping point that would (1) increase access to care; (2) accelerate training and research on the prevention and treatment of obesity; and (3) reduce weight stigma.

Ten years later, obesity endures as one of our country’s greatest public health issues. While the past decade has witnessed some advances with respect to obesity treatment, training, and weight stigma, there is unrealized potential that awaits attention. Below, we discuss major developments and areas of unrealized potential in these three domains. While many of these developments cannot be directly attributed to the AMA’s classification of obesity as a disease, they are nonetheless critical for understanding shifts that have occurred in the obesity public health landscape since the AMA’s decision and for identifying avenues for future progress toward more accessible, equitable, and high-quality obesity care.

## 2. Access to care

Several notable advancements in access to obesity care have occurred in the last 10 years. The three primary, evidence-based approaches to treatment are lifestyle modification (through decreased caloric intake, increased physical activity, and instruction in behavioral modification), FDA-approved anti-obesity medications, and bariatric surgery (5).

In the past decade, and particularly during the lockdown necessitated by the COVID-19 pandemic, lifestyle modification approaches have largely moved from in person delivery to eHealth/mHealth based approaches using web portals, text messaging, telephone counseling, or web conferencing to deliver care. These remote forms of treatment delivery reduce several logistical hurdles such as travel time and costs as well as the need to be located geographically close to providers (6). Dissemination of evidence-based lifestyle modification programs into community-based settings has moved care from behind the walls of academy and specialized weight management centers to more accessible points of contact.

Despite these developments, annually less than 5% of Americans participate in a formal weight loss program of any type (7). Many insurance companies still do not cover participation in lifestyle modification programs. Even if individuals do have insurance that covers interventions, patients can be hard-pressed to find available treatment programs and providers (8). Insurance benefit design, which often includes strict stipulations around who can provide lifestyle modification; undervaluation of the impact of treatment; and a lack of trained providers to deliver care further contribute to limited access (9, 10).

Among those who do participate in lifestyle modification programs, there is significant heterogeneity in response to treatment (5). Weight recurrence (i.e., weight regain) after intentional weight loss remains a significant challenge. While, on average, patients lose 5%–7% of their weight within 6 months of treatment, the vast majority of individuals regain most of this weight overtime (5). Further, participants from minoritized and under resourced groups have been underrepresented in the lifestyle modification evidence base, resulting in low external validity. When these programs have been scaled to these groups, many of whom are disproportionately affected by obesity, weight losses are typically smaller (11).

In the last few years, several new pharmacotherapy agents have received FDA approval. The efficacy of the most recent generation of these medications far outpaces previously available options (12). For example, in recent randomized trials, over 70% of participants taking the medication semaglutide lost at least 10% of their body weight (12). Several additional medications with similarly impressive outcomes are in the pipeline for FDA approval. In short, the landscape of anti-obesity medications is dramatically different than it was 10 years ago, both in terms of the range of available options and their efficacy.

While we have arguably entered a new phase of possibilities for pharmacotherapeutic treatment of obesity, anti-obesity medication use remains low (1%–3%) (13). Multiple drivers contribute to low usage. Cost is a significant barrier, particularly for the newer anti-obesity medications, which have an exorbitantly high out-of-pocket cost in the United States of over $1,000/month. While a few insurers cover these newer medications (14)—a development that seems unlikely to have occurred had the AMA and other similar organizations not recognized obesity as a disease—the vast majority do not, meaning interested patients are left no choice but to pay out-of-pocket, try lower cost alternatives, or defer treatment. Additionally, as is the case with medications for most chronic conditions, medication use needs to be sustained to provide continued benefit. Thus, the long-term financial costs of staying on the newest medications is prohibitive for many.

Many of the previously approved medications have a much lower cost. While the efficacy is not as high as the latest generation of medications, the cost effectiveness suggests that prescribing should be much higher than current levels. For example, phentermine/topiramate extended release is available for less than 1/10th of the cost of the newest anti-obesity medications, and more than 50% of patients on this medication lost at least 10% of their body weight (12). Ignoring lower-cost treatment options for those with fewer resources threatens to widen existing disparities in obesity. Many factors may impact initiation and long-term use of anti-obesity medications, likely including many patients, providers, and third-party payers not viewing obesity as a disease and thus being hesitant to use medications for obesity like they would for other chronic diseases. It is also clear that more work is needed to understand how maintenance of a weight reduced state can be cost-effectively sustained with pharmacotherapy.

Bariatric surgery offers the most substantial and long-lasting weight loss and health improvements of available obesity treatments. The most common procedures produce average weight losses of approximately 30% at 1 year, with weight losses of 20% or more sustained by many individuals throughout the first postoperative decade (12). These weight losses are associated with significant improvement in obesity related comorbidities such as type 2 diabetes, hypertension and heart disease, as well as reduced risk of multiple forms of cancer (15). Given amassing evidence for the long-term health benefits of bariatric surgery, a new set of patient eligibility guidelines was released this past year that recommended lower body mass index and age thresholds for who should be considered for surgery (15). These guidelines are expected to further expand access to care.

Despite bariatric surgery’s effectiveness, only 1% of eligible individuals receive surgery (16). One major unaddressed treatment barrier is insurance benefit design (9). Although most major insurance companies now cover bariatric surgery, some, like Medicaid, require that surgery be performed at a Bariatric Center for Excellence or have other precertification criteria that limits access. Underinsurance, as evidenced by plans with high cost-sharing, further hinders utilization of bariatric surgery (9). A significant portion of patients who are eligible for surgery lack health insurance altogether. This highlights the disproportionate impact of obesity on segments of the population most adversely affected by social determinants of health, as well as the powerful influence of upstream factors on treatment access. Indeed, individuals identifying as Black, those with lower incomes, and those with non-private insurance are less likely to have bariatric surgery (17).

## 3. Training and prevention

There are signs that the training in the etiology and treatment of obesity for a range of health care disciplines has begun to increase. Professional societies and expert working groups have developed competencies and benchmarks for obesity-focused training for both medical students and other health providers (18). Several training strategies have shown some effectiveness in improving medical trainees’ obesity-related knowledge, attitudes, and skills (19). Additionally, more physicians are pursuing board certification in obesity medicine. The number of board-certified physicians has increased more than tenfold in the past 10 years and certified providers exist in all 50 states (8).

Still, training in obesity is not where it needs to be. The number of medical schools that include comprehensive obesity medicine education in their curriculum remains shockingly low and most physicians do not seek specialized training in obesity at the postgraduate level (10, 20). Obesity training for allied health professionals (e.g., medical assistants, dietitians) and community health workers—all of whom could play a key role in helping to manage obesity in both clinic and community settings—is similarly underdeveloped (10).

There are multiple negative effects of inadequate provider training. For example, under-trained providers are less likely to screen for, diagnose, or provide prevention and treatment as recommended per clinical guidelines (21). Many providers hold inaccurate beliefs about obesity, lack knowledge of prevention and treatment options, and report being uncertain of how to discuss obesity with their patients (10). Presumably in part because of this, patients, too, have knowledge gaps about their own weight status and treatment options, further increasing the likelihood that they do not receive high quality, evidence-based care (21).

Over the last decades, many efforts have focused on obesity prevention among youth. This is critically important work, given that obesity early in life not only confers immediate health risks but is also a strong precursor for obesity and related comorbidities in adulthood. A variety of preventative approaches have shown moderate efficacy. These include school-based programs, government-sponsored programs that help to ensure access to healthy foods, policy-based initiatives, family-focused lifestyle modification interventions, and multilevel interventions (22). Given continued research highlighting the important role that factors like the built environment, food insecurity, stress, and structural racism play in increasing risk for obesity, there have also been increased calls to address social determinants of health to help prevent obesity across the lifespan and reduce related health disparities (23).

Notwithstanding this progress, it is hard not to conclude that prevention efforts, taken collectively, have had limited success in stemming the growth of obesity in American children. They also have not reduced racial and ethnic disparities seen in obesity. Due to the strong influence of social determinants of health on obesity risk, change at the policy, environment, and systems level is needed to achieve more effective and equitable obesity (23).

## 4. Weight stigma

Weight stigma refers to social devaluation of people because of their body weight (24). Weight stigma has a range of deleterious effects, not the least of which includes contributing to further weight gain via both physiological and behavioral pathways (24).

Encouragingly, the issue of weight stigma has received more attention in the past 10 years and there have been efforts to reduce weight stigma at several levels. For instance, there has been a purposeful shift within the medical and research community to use terminology that reflects obesity being a disease rather than language implying obesity is a personal choice. Examples of this include using person-first language (e.g., “individuals with obesity” rather than “obese individuals”) and using the term “weight recurrence” rather than “weight regain” to better reflect the chronic nature of obesity. The field also has moved away from value-laden terms like “weight loss success” and “weight loss failure” when describing treatment responses. More recently, some have even advocated for modifying the approach to coding for obesity in medical records with the term “adiposity-based chronic disease” (25). At present, the field remains challenged to reach consensus with the use of these terms.

Several strategies have shown modest efficacy for reducing weight stigma in healthcare settings (26). Awareness of, and advocacy against, weight stigma has also increased among segments of both the public and the scientific community. Multidisciplinary groups of experts have drafted excellent statements describing the importance of ending weight stigma and providing roadmaps for doing so (27, 28).

Despite these steps forward, weight stigma remains a tremendous problem. Weight stigma is just as pervasive in the United States as it was 10 years ago. Indeed, weight stigma is seen among individuals of all ages, backgrounds, and health statuses (28). Individuals with obesity are frequently the subject of bias and discrimination in educational, work, and healthcare settings. The negative effects of this bias and discrimination should not be understated. For example, weight bias in healthcare settings can lead to mistrust of providers, the delay or avoidance of healthcare, and poorer health outcomes (24).

Weight discrimination—one consequence of weight bias—remains legal at the federal level and in all but one state (27). Similarly, policies to protect youth from weight-based teasing are rare (27). While, as noted above, interventions to address weight stigma at the individual level show small positive effects, multilevel approaches to address weight stigma are sorely lacking.

## 5. Discussion

Did the changes in the obesity prevention and treatment landscape that many hoped for when the AMA classified obesity as a disease come to fruition? Certainly, progress has been made. Yet, we are unaware of any change in policy or law that can be directly tied to the AMA’s classification of obesity as a disease, and significant work remains to create equitable access to evidence-based treatments, bring research and training on obesity on par with its prevalence, and reduce the pervasiveness and harm of weight stigma. We believe there are several key steps to advance these goals:

- Reduce systematic barriers to treatment access. This includes ensuring insurance coverage for multimodal treatment approaches, designing coverage to maximize individuals’ ability to engage with treatment and to remove non-evidence-based eligibility hurdles, and increasing provider reimbursement for obesity treatment to encourage wider availability.
- Embrace a chronic disease care model for obesity that provides a range of high-quality, evidence-based intervention strategies at all levels of care, from community to primary care to specialty care.
- Continue support for research on the mechanisms underlying obesity, multifactorial contributors to obesity, and multilevel prevention and treatment approaches. Given disparities in obesity prevalence and impact, health equity should be a top priority in these efforts.
- Enact additional policies, environmental changes, and systemic changes to address social determinants of health and upstream contributors to obesity. This work should be deeply rooted in principles of health equity. Frameworks, such as that described by Kumanyika (23), can inform this work.
- Increase the prioritization of training in obesity medicine for both trainees and licensed providers across a range of specializations. Benchmarks and training approaches developed since 2013 can guide these efforts. To maximize benefit and guard against harm, training in obesity medicine should go hand in hand with training in weight stigma and eating disorder prevention, screening, and treatment.
- Implement a comprehensive, multilevel agenda for ending weight stigma. This agenda should include legislative change, reframing of the public health obesity narrative to align it with scientific understanding of obesity, logistical and cultural changes to reduce stigma in healthcare, and more. Several outstanding pieces have been written on recommendations for reducing weight stigma and its harm (28).
- Create an environment in which patient-centered, compassionate, nonjudgmental medical care for obesity is the norm. Treatment decisions should respect patients’ values and autonomy and stem from shared decision-making. Improved patient education on obesity and evidence-based prevention and treatment options can better equip patients for informed decision-making. A non-weight centric approach may provide benefit for those uninterested in obesity-focused treatment.

Although the scope, and importance, of the remaining work to be done looms large, we remain optimistic about the future. Truly conceptualizing and treating obesity as a chronic disease requires a major paradigm shift at both the public health and individual health level, and this work is young. Ten years from now, we hope to reflect back on a much-improved obesity public health landscape.

## Data availability statement

The original contributions presented in the study are included in the article/supplementary material, further inquiries can be directed to the corresponding author.

## Author contributions

LS conceived of the piece, with input from DS. LS wrote an initial version of the manuscript. DS and JA revised the manuscript. All authors contributed to the article and approved the submitted version.

## Funding

This manuscript was supported by a Loan Repayment Award from the National Heart, Lung, and Blood Institute (recipient: LS; L30HL154167).

## Conflict of interest

LS reports receiving a Loan Repayment Award from the National Heart, Lung, and Blood Institute. DS currently has grant funding from the National Institute of Dental and Craniofacial Research, National Institute for Diabetes, Digestive, and Kidney Disease as well as the Department of Defense. He has consulting relationships with Ethicon, Novo Nordisk, and Twenty30 Health. JA reports research funding from Nestle Healthcare Nutrition, Eli Lilly, Boehringer Ingelheim, Epitomee, Inc., UnitedHealth Group R&D, KVKTech, and WW. JA also reports serving as a consultant for Nestle Healthcare Nutrition, Eli Lilly, Optum Labs R&D, Novo Nordisk, Spokes Health, Inc., Intuitive, Regeneron, and Brightseed, as well as serving on the advisory board for Novo Nordisk, Nestle Healthcare Nutrition, Eli Lilly, Level2, and WW.

## Publisher’s note

All claims expressed in this article are solely those of the authors and do not necessarily represent those of their affiliated organizations, or those of the publisher, the editors and the reviewers. Any product that may be evaluated in this article, or claim that may be made by its manufacturer, is not guaranteed or endorsed by the publisher.
